# Establishment of an AI-supported scoring system for neuroglial cells

**DOI:** 10.3389/fncel.2025.1584422

**Published:** 2025-06-19

**Authors:** Annika Bitsch, Manfred Henrich, Svenja Susanne Erika Körber, Kathrin Büttner, Christiane Herden

**Affiliations:** ^1^Institute of Veterinary Pathology, Justus Liebig University Giessen, Gießen, Germany; ^2^Center of Mind, Brain and Behaviour, Justus Liebig University Giessen, Gießen, Germany; ^3^Biomathematics and Data Processing Group of the Department of Veterinary Medicine, Justus Liebig University Giessen, Gießen, Germany

**Keywords:** neuroglial cells, neuronal projections, microglia, astrocytes, artificial intelligence based scoring systems, morphological complexity, hippocampal slice cultures

## Abstract

The feasibility of a computer-aided scoring system based on artificial intelligence to detect and classify morphological changes in neuroglial cells was assessed in this study. The system was applied to hippocampal organotypic slice cultures (OHC) from 5 to 7 day-old wild-type and TNF-overexpressing mice in order to analyze effects of a proinflammtory stimulus such as TNF. The area fraction of cells, cell number, number of cell processes and area of the cell nucleus were used as target variables. Immunfluorescence labeling was used to visualize neuronal processes (anti-neurofilaments), microglia (anti-Iba1) and astrocytes (anti-GFAP). The analytic system was able to reliably detect differences in the applied target variables such as the increase in neuronal processes over a period of 14 days in both mouse lines. The number of microglial projections and the microglial cell number provided reliable information about activation level. In addition, the area of microglial cell nuclei was suitable for classification of microglia into activity levels. This scoring system was supported by description of morphology, using the automatically created cell masks. Therefore, this scoring system is suitable for morphological description and linking the morphology with the respective cellular activity level employing analyses of large data sets in a short time.

## 1 Introduction

Scoring systems are already used to characterize the morphology of neuroglial cells. Traditionally, a manual procedure was used to determine cell number, e.g., by counting cells using optical grids and a hand counter (Lovelace and Cahill, 2007). Due to the heterogeneity of neuroglial cell morphology, unambiguous cell identification is difficult. Computer-aided methods are one way of circumventing these difficulties. The addition of algorithms for automatic cell counting is often combined with programs that can recognize shapes (ZEISS software apeer, open source software ImageJ) (de Gracia et al., 2015). Cells can be labeled by respective specific expression of antigens that can be visualized by immunofluorescence microscopy, e.g., using Apo Tome (Carl Zeiss Microscopy GmbH, Oberkochen, Germany) (Crews et al., 2004; Nixon et al., 2008; Oberheim et al., 2008; Yokose et al., 2011). The hippocampus represents an advantageous brain region because region-specific temporal changes can be documented in a reliable manner (Hailer et al., 1999; Zhao et al., 2003). By using organotypic hippocampal tissue cultures, it is possible to maintain three-dimensional tissue organization *in vitro* (De Paola et al., 2003; Gähwiler, 1981; Gähwiler et al., 1997; Galimberti et al., 2006; Stoppini et al., 1991) and terms of the 3R principle are addressed (Galimberti et al., 2006; Stoppini et al., 1991). The hippocampal formation consists of four cortical regions. These include the dentate gyrus as well as the cornu ammonis itself, which can be divided into three subunits (CA3, CA2, CA1) (Amaral, 1978). Even more, the hippocampus contains numerous conduction pathways, which are aligned in transverse and septotemporal axes (Caroni, 1997; De Paola et al., 2003; Feng et al., 2000; Galimberti et al., 2006). Conduction pathways formed by neuronal projections are predominantly composed of the intermediate neuronal filament neurofilament (Franke, 1987; Steinert et al., 1985). Accordingly, neurofilaments are components of the basic structure of axons as well as dendrites (Droz et al., 1973; Hoffman and Lasek, 1975; Schlaepfer, 1971) and are therefore widely used to mark these processes.

Astrocytes as another large neuroglial population can be labeled based on the expression of their cell specific marker GFAP (glial fibrillary acidic protein). This makes it possible to determine the diameter of the cell, the length and thickness of the astrocyte processes, and even their number. The diameter of the astrocyte is determined by the longest line running through the cell, which also crosses the nucleus whereas the length of a process is defined as the distance between the nucleus and the tip of the process, with the width of the process measured at two opposing GFAP mark points (Oberheim et al., 2008). For microglial cells a threshold method that correlates the cellular morphology to their activity state has already been established (Kozlowski and Weimer, 2012). In this method, the size of the soma, the cell circumference as well as length and the surface area of the cell were quantified. A method for the evaluation of the morphology of microglia, which was already used in previous studies of our group, is based on the principle of Hovens et al. (2014). The aim was to draw conclusions about the functionality status of the microglia based on the morphology. The evaluation was performed on digital images with the program “Fiji is just ImageJ”^1^ to set the threshold for the microglia (Onkels, data not shown). Results for cell size, cell body size (i.e., without processes), and number of microglia were correlated to assign microglial morphology to a state of activity (Hovens et al., 2014). Microglia are activated by proinflammatory mediators such as TNF (tumor necrosis factor) (Takeuchi et al., 2006; Verkhratsky and Kirchhoff, 2007), causing them to transition from an inactive ramified to an activated amoeboid state. Thus, the morphology of neuroglial tissue is influenced by the activity status of these cells (Faustmann et al., 2003; Hinkerohe et al., 2005). Inflammatory processes in the CNS are regularly accompanied by activation and proliferation of glial cells, which are activated by the biochemical environment (Kettenmann et al., 2011). These proinflammatory substances also include TNF (Merbl et al., 2014; Smeal et al., 2012). To visualize the neuroglial alteration triggered by the proinflammatory cytokine TNF, the use of mice with an altered TNF system has been proven suitable (Hirz, 2017; Kramer et al., 2012; Vienenkoetter et al., 2015; Osei et al., 2024). Here, the effect of proinflammatory preconditioning in TNF-overexpressing mice (TNF.MK.41.3) and respective morphological changes in neuroglial cells can be observed and compared to wild-type (wt) mice (Hirz, 2017; Kramer et al., 2012). Thus, this model system was chosen to establish and be applied for new AI-based scoring system for neuroglial cells employing the hippocampus as model region.

For this AI-based scoring system, the use of digital image data with Z-stacks and subsequent generation of maximum intensity projections of the selected planes allows to transform a 3D image into a 2D projection, which can be used for further segmentation of cells. Depending on the penetration depth of the immunofluorescence marker used and the slice width, a different slice depth can be used. It is possible to examine multiple partial Z-volumes from one slice (Kozlowski and Weimer, 2012) or, for example, to approximate the cell volume by scanning one plane after the next, which belong to a Z-stack, horizontally separately (Plog et al., 2014). However, if a 3-dimensional structure is intersected by a plane, its intersection is 2-dimensional (Elias et al., 1971). This results in the following problems for certain target sizes, e.g., the area of objects that can be determined in only one section plane is affected by the orientation of the objects in space. For non-symmetric objects, the areas can differ significantly depending on the section plane. Furthermore, the curvature of the surface is crucial for the acquisition of the volumes. If the spacing of the planes along the Z axis is chosen too large, minimal curvatures may be lost (Elias et al., 1971).

The goal of this study was to develop an unbiased AI-based scoring system for neuroglial cells enabling to relate the morphology of different neuroglial cells to their functionality. This was achieved by analyzing areas of neuronal processes, astrocytes and microglia as well as activity status of the microglia in the hippocampus of TNF overexpressing and wt mice as model system.

## 2 Materials and methods

Immunofluorescence images of the CA3 region were recorded on hippocampal slice cultures of 5–7 day old mice. The fixation time points of the OHCs were day 3, 7, and 14 after preparation of the mice hippocampi. The target cells were marked subsequently by using cell masks in order to develop an AI-supported scoring-system.

### 2.1 Mouse model

For method establishment and testing, two mouse strains were used at 5–7 days of age without considering sex distribution. The mouse strains are based on a C57BL/6J background. The breeding of these mouse strains was localized in the central animal laboratory of the Justus Liebig University Giessen. In accordance with the Animal Welfare Act and the principles of the 3Rs (Replacement, Reduction, Refinement), the animals were killed and the organs obtained (JLU number 756_M) to produce OHCs with which the experiments were carried out. The wild-type mice (C57BL/6JOlaHsd) were bred by Harlan Laboratories GmbH. The TNF.MK.41.3 mice (C57Bl/6-Tg(Grin2b-Tnf)41.3MK) were kindly provided by Prof. Dr. U.L.M. Eisel, Department of Molecular Neurobiology, University of Groningen, Netherlands. The transgenic status of the mice was determined using methods as previously reported (Kramer et al., 2012).

### 2.2 Organotypic hippocampal slices and immunofluorescence staining protocol

Organotypic hippocampal slice cultures (OHCs) were prepared based on the method reported by Stoppini et al. (1991). The brain was placed in a specific manner so that the medians pointed upward and the bulbus olfactorius pointed cranially. For further preparation of the midbrain, the original protocol (Galimberti et al., 2006) was modified.

**FIGURE 1:**
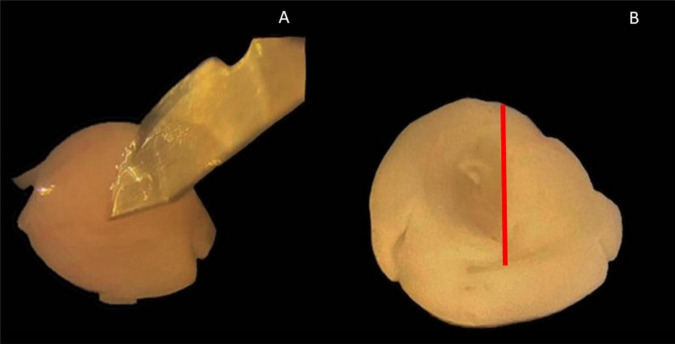
Hippocampus isolation using a scalpel. **(A)** Scalpel points to incision with intact cerebral hemisphere with a meadian view. **(B)** View of medians after detachment of the midbrain; red line: incision line at which the midbrain was detached.

A scalpel incision was made through the midbrain at the level of the third ventricle. The length of this cut was 2/3 of the vertical of the brain and ended at the level of the cerebral cortex (Figure 1). The fimbria hippocampi was also transected to facilitate the excision of the hippocampus. Once the midbrain was detached, the hippocampus was visible and detached laterally. Slices were now generated according to the previous protocol (Galimberti et al., 2006). The hippocampus cut by a tissue chopper (McIlwain Tissue Chopper model TC752) into 350 μm thick slices. The hippocampal tissue cultures (slices, OHC) were then placed into an incubator at 37°C, humidity of 90% and CO2 content of 5%.

To ensure survival of OHC, nutrient medium was changed on days 3 and 7 after dissection. OHCs maintained in culture until day 14 and were stained according to the principle of Galimberti et al. (2006). Fixation took place on days 3, 7, and 14 after hippocampal preparation. To achieve permeability of the tissue sections, 1 mL of 0.8% Triton X-100 in PBS was pipetted over and 1 ml next to the insert, in deviation from the initial protocol. This preparation incubated for 12–18 h at 4°C. Subsequently, the primary antibody was added in the designated dilution, which also contained 1% Triton X-100, in a volume of 90 μl on the OHC located in the well and was incubated overnight at 4°C. Neurofilament anti mouse antibody 1:500 (M0762 Dako, Hamburg, Germany) was used to label neuronal processes, GFAP anti guinea pig antibody 1:1,000 (173004 Synaptic Systems GmbH, Göttingen, Germany) was used for astrocyte labeling, and Iba1 anti rabbit antibody 1:1,000 (01919741 Fujifilm Wako Chemicals Europe GmbH, Ratingen, Germany) was used to visualize microglia. Nuclear labeling was classically performed with Dapi at a dilution of 1:400 (Carl Roth GmbH und Co., KG, Karlsruhe, Germany). Alexa 647 Donkey anti guineapig (1:200, Dianova GmbH, Hamburg, Germany), Alexa 488 Donkey anti rabbit (1:200, Thermo Fisher Scientific Inc., Waltham, MA, United States) and Alexa 594 Donkey anti mouse (1:100, Thermo Fisher Scientific Inc., Waltham, MA, United States) served as secondary antibodies. Also added to the dilution was 1% Triton-X-100. To reduce number of OHCs, staining of microglia and astrocytes was performed as double staining.

#### 2.3 Evaluation of the immunofluorescence images

**FIGURE 2:**
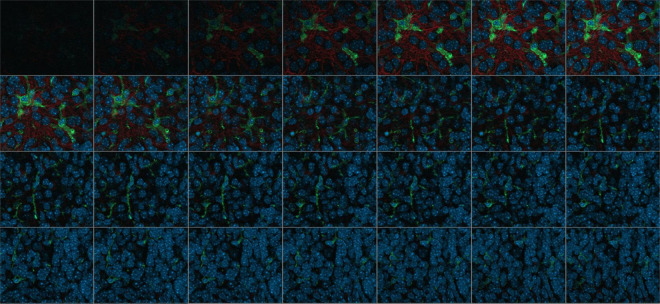
Single images of a Z-stack consisting of 28 images, Wild-typ: red: GFAP-labeled astrocytes; green: Iba1-labeled microglia; blue: Dapi-labeled nuclei.

Immunofluorescence was evaluated on images acquired using the Zeiss-Axio Observer 7 ACR immunofluorescence microscope (Carl Zeiss Microscopy Deutschland GmbH, Oberkochen, Germany). The images were taken using reflected light fluorescence in combination with Colibri lights. OHCs were automatically detected on the slide using the “AI sample finder” module (Figure 2).

Computer-aided analysis was performed with the program ZEN 3.6. The measurement procedures were automated by the modules “Intellesis Segmentation” (ZEISS) and “Apeer” (ZEISS). The latter is based on a machine learning algorithm for object marking and automatic image segmentation. Object and background marking were performed under visual control. For this purpose, Z-stacks consisting of up to 30 single images were used and were merged to a maximum intensity projection. The individual planes were spaced 0.24 μm along the Z axis (Figure 2).

Subsequently, a quality control of the developed image masks was carried out, which were performed separately for neuronal processes, astrocytes and microglia. The quality control included comparison of immunofluorescence labeling with the image mask and the background error. Therefore, the evaluation of immunofluorescently labeled cells was performed under visual control. Only images of sufficient signal strength were included in the analysis.

**FIGURE 3:**
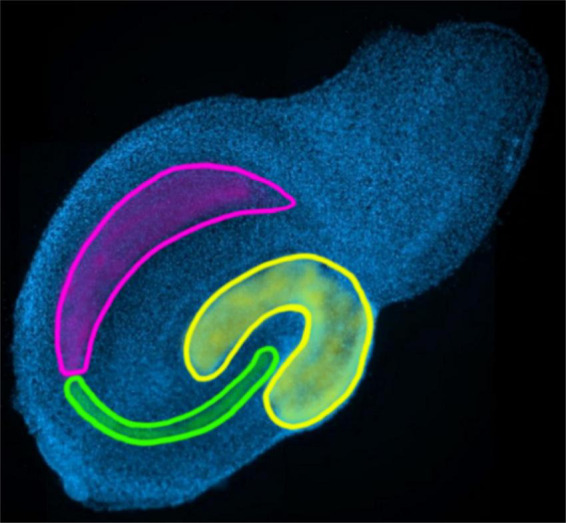
Marking of the regions of the hippocampus to be evaluated: yellow: dentate gyrus; green: CA3; pink: CA1-2 in the overview image of the AI-sample-finder.

To test the established and controlled method, a known TNF-based change in neuroglial cells was examined on wild-type and TNF.MK.41.3 mice. In an application, the algorithm was tested to see if significant differences of cell morphology could be detected. The effect of the mouse line was examined for each cell line. Data sets were divided according to the hippocampal region CA3 and additionally according to the time of fixation (days 3, 7, and 14) of OHCs (Figure 3).

**FIGURE 4:**
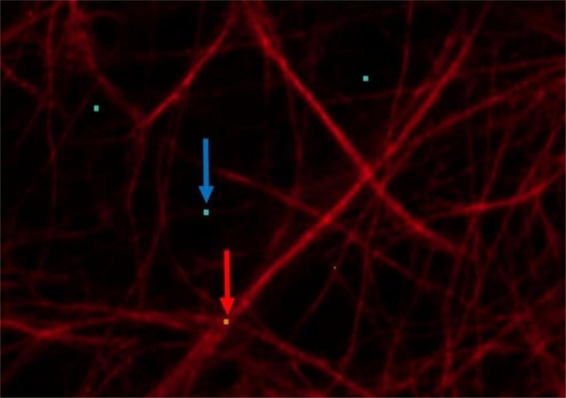
Astrocytes (red) displayed by GFAP: red arrow: points to orange pixel for object marking; blue arrow: points to light blue pixel for background marking.

**FIGURE 5:**
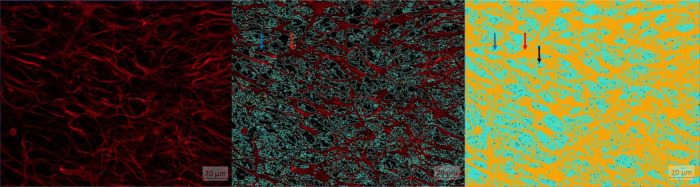
AI training results. **(A)** Image basis: astrocytes visualized using GFAP. **(B)** Astrocytes visualized using GFAP outlined with light blue line for object representation: red arrow: object (astrocytes), blue arrow: background. **(C)** Astrocytes: red arrow: object orange (astrocytes), blue arrow: background light blue, black arrow: pixel groups below threshold (= 24).

The following main target variables were set to study the neuroglial network: The number of projections should be determined in addition to cell number and cell size. Here, the percentage area of neuronal projections, astrocytes and microglia was used. The basis of this area calculation was formed by five images, whereby object and background recognition were trained by means of “Intellesis Segmentation.” For this purpose, the objects (neuronal processes, astrocytes and microglia) and the background were marked manually (Figure 4). Therefore, the evaluation of immunofluorescently labeled cells was performed under visual control. The pixel colors were assigned to the two categories accordingly. To reduce the background error (false positive signal), a threshold was set, which consisted of a certain number of pixels. Thus, pixel groupings below the threshold were not included in the evaluation (Figure 5).

The results of the training formed the basis for the evaluations. The following figures (Figure 5) show the image basis and the color-marked area calculations determined by the program exemplarily for astrocytes. The same cell mask was used for neuronal process and microglia. Thus, the correctness of the signal was checked while the measurement program was calculating the area fraction, without having to schedule time for this again.

**FIGURE 6:**
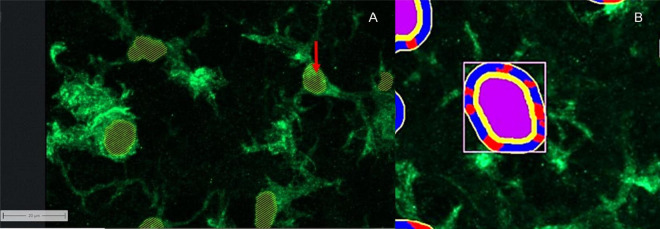
Computer software working together. **(A)** Cell nucleus labeling of microglia using apeer: red arrow: points to the labeling of the cell nuclei. **(B)** Allocation of image information to different categories using ZEISS ZEN Intellesis: Microglia displayed using Iba1: purple area: cell nucleus; yellow area: border area around the defined cell nucleus with defined number of pixels; red area: extensions defined by the program; blue area: background in the defined border area.

In order to assess the functional changes and activity status of microglia more precisely, the software module “apeer” was used to develop a method that identifies the cell nuclei (Figure 6A). The criterion that a microglia nucleus had to fulfill was an Iba1 marked (green) border around the nucleus and the nucleus itself, which had blue pixels.

The cell nucleus labeling (apeer) in combination with the microglia area (Intellesis segmentation) became the basis of another evaluation procedure, with the aim to identify the number of microglia cell nuclei, the area of cell nuclei and the number of projections per cell. For this purpose, it was necessary to use the zone of influence measurement. Here, the cell nucleus (primary object) was surrounded by a ring in which the cell processes were seen (Figure 6B). The distance between the ring and the primary object (yellow) was 9 pixels. The width of the ring corresponds to 14 pixel. The microglial processes are defined as contiguous green pixels marked by anti-Iba1 antibody, which are counted in the ring. If the green pixels are separated by black ones, they are perceived as separate processes. Two different masks were used to represent the nuclei and the ring. On the one hand, the microglia and the zone of influence were outlined by lines, on the other hand, a mask could be chosen which marked the zones of influence.

These measurement procedures were quality controlled by describing the astrocytes, microglia and neuronal projections. This specially performed optical quality control compared the observed morphology of the neuroglial cells with the calculated parameters. The cell parameters are the area fraction of the neuronal processes, the astrocytes and the microglia and the number of microglia processes, the microglia cells, as well as the area fraction of the microglia cell nuclei. The aim was to determine whether the morphology detected by the algorithm adequately described the cell.

### 2.4 Statistical analysis

Statistical analysis was performed by the Biomathematics and Data Processing Group of the Department of Veterinary Medicine, Justus Liebig University Giessen, using SAS 9.4 (SAS^®^ Institute Inc., 2013. Base SAS^®^ 9.4 Procedures Guide: Statistical Procedures, 2nd edition ed. Statistical Analysis System Institute Inc., Cary, NC, United States). The results of immunofluorescence examination of the hippocampus region CA3 were used as the data basis. The time points 3, 7, and 14 after preparation were taken into consideration. Here, the mean value of each image to be evaluated was calculated for each mouse at each hippocampal region and at each time point. Up to 9 mice were evaluated per parameter composition. This was used for further analysis.

The data was first tested for normal distribution. If the data set was normally distributed, the mean values were used. In the case of a data set that achieved normal distribution only after logarithmization, the logarithmized values were added in brackets. Thus, OHCs were analyzed separately by region and days in culture for the effect of mouse line. Here, a pairwise comparison between the wild-type and TNF-overexpressing mice was subsequently performed. In addition, the time course was analyzed.

## 3 Results

The following main target variables were established to study the neuroglial network: the percentage area of neuronal projections, astrocytes and microglia. Additional parameters were established to characterize microglia more precisely such as cell number, number of microglial projections and nucleus size and activity status.

### 3.1 Neuronal processes: morphological characterization and percentage area

**FIGURE 7:**
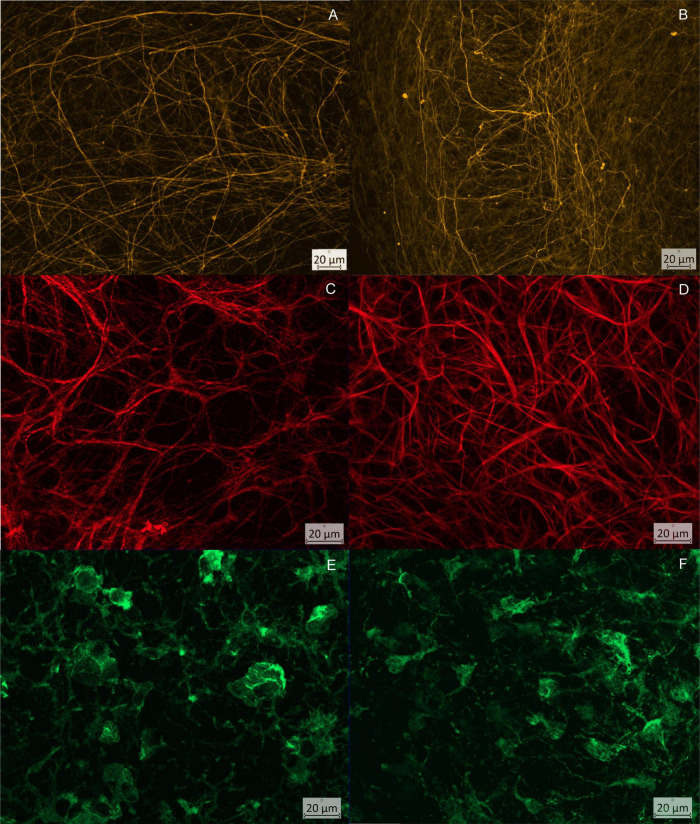
Comparison of the CA3 region of wild type (column 1) and TNF.MK.41.3 (column 2) at defined time points: **(A,B)** neurofilaments visualized using Neurofilament, time point = day 3; **(A)** clearly recognizable parallelism of the cross struts through the pyramidal cell layer; **(B)** dense network of neuronal projections, heterogeneous arrangement and parallel course of the cross struts through the pyramidal cell layer; **(C,D)** visualization of astrocytes using GFAP, time point = day 7; **(C)** loose network of fine projections that sort into bundles to a small extent; **(D)** dense network of astrocytes consisting of fine projections with bundling of several projections and formation of broad streets; **(E,F)** visualization of microglia using Iba1, time point = day 14; **(E)** microglia with 3 or more projections, long branched projections; **(F)** reduced cell number, with broad short projections.

Qualitative assessment performed by the human observer revealed several structural abnormalities of the segmented maximum projections. It became clear that the arrangement of the neuronal projections by comparing the wild-type and the TNF-overexpressing mice in space changed. On the one hand, the neuronal projections traversed the pyramidal cell layer with parallel transverse struts. Orthogonal to these parallel projections, the pyramidal cell layer was framed by multiple projections along its course (Figure 7). This arrangement always found into the hippocampus of the wild-type mice. The arrangement of the neuronal processes of TNF-overexpressing mice differs in order of parallel and orthogonal ordered processes that are joined by transverse and additional processes, rather be described as irregular.

At all-time points, OHCs of TNF-overexpressing mice were found to have a dense neuronal network with broad neuronal projections and increased branches projecting into the pyramidal layer from the outside. The basic structure of the parallel framing neuronal projections was obscured by heterogeneous disorderly arranged projections (Figures 7A,B). This fits to the increased area fractions of neuronal projections per total image at day 7 and 14 (Table 1) in the TNFTg mice OHCs when comparing to the wt mice. Furthermore, an increase in the area fraction of axons and dendrites could be detected in this region and in both mouse lines compared days 7 to 3. The mouse line also had a significant effect on the area fraction of neuronal processes at day 14 after preparation in CA3 (*p* = 0.0032). A reduction in the area fraction of axons and dendrites was found at both mouse lines from days 7 to 14. Furthermore the TNF.MK.41.3-mice showed a significant larger percentage area of the neuronal projections on days 7 and 14 than the wild-type mice (*p* = 0.0053) (Table 1). From the comparisons of the area fractions of the neuronal processes, it was evident that the area fraction of the total image area in the TNF-overexpressing TNF.MK.41.3 mice was larger (43.78%) than that of the wild-types (31.09%) at day 7. On day 14 the area fraction of the total image area TNF-overexpressing TNFMK41.3 (36.57%) mice was larger than that of the wild-types (19.39%).

**TABLE 1:** Neuronal processes: percentage area.

Mouse line	Time	CA3
Wild type	Day 3	33.77
	Day 7	31.09
	Day 14	19.39
TNF.MK.41.3	Day 3	32.47
	Day 7	43.78
	Day 14	36.57

### 3.2 Astrocytes: morphological characterization and percentage area

The astrocytes within a maximum projection of a wild-type hippocampus formed a network of many thin processes, which ran as a fine-mesh or in parallel paths through the pyramidal cell layer at every time point (Figure 7C). Multiple long thin projections were arranged either as bundles or form isolated wide roads. Bundling increased from days 3 to 14. Thus, a heterogeneous network was present at day 14 after dissection, which seems to be composed of different morphological subpopulations of astrocytes (Emsley and Macklis, 2006). On the other hand, an increase in the number of processes or hyperplasia may also be responsible for the increase in area and thus the activation of astrocytes.

These broad roads were also observed in the TNF-overexpressing mice. The spatial arrangement of the projections remained the same as in the-wild types, but an increase in the area of the astrocytes was measured compared to the wild-types at day 7 (Figure 7D).

At day 3 after dissection, the mouse line had no significant effect (*p* = 0.1326) on the area fraction of astrocytes in region CA3. In contrast, at day 7 after preparation a significant effect of the mouse line was detected on the area percentage of astrocytes in the CA3 region (*p* = 0.0497) with TNF.MK.41.3 possessing twice the area percentage compared to wild-type. However, at day 14 after dissection the mouse line had no effect on the area percentage of astrocytes in the CA3 region from the total image area (*p* = 0.1396) (Table 2). An increase in the percentage area of astrocytes in the TNF.MK.41.3 mice on day 7 results from the observed increase in projections and cell body size of astrocytes in the TNF.MK.41.3 mice.

**TABLE 2:** Astrocytes: percentage area.

Mouse line	Time	CA3
Wild type	Day 3	32.36
	Day 7	21.51
	Day 14	50.91
TNF.MK.41.3	Day 3	32.45
	Day 7	43.83
	Day 14	32.74

### 3.3 Microglia: morphological characterization and percentage area

During the establishment of the algorithms, the heterogeneity of the microglia morphology was striking. For example, cells of different sizes, cell bodies, and projections were detected. Several differently shaped microglia were observed simultaneously on a maximum intensity projection. The differences in morphology were due to the length and width of the microglia processes, as well as the size of the cell nucleus. Due to the mostly isolated location of cell bodies, several parameters were established to classify the microglia. First, the parameter of percentage area measurement was applied similarly as for the neuronal processes and astrocytes. To assess the complexity of microglia and their activity state, several parameters were used additionally for their characterization.

By displaying the 3D image in a 2D intensity projection, stereology must be taken into account. Using microglia as an example, it was shown that the Z-planes can intersect the cells at different levels. Only the addition of these to a maximum projection minimizes the error of not fully capturing the structure of the cell. Thus, in this work, the section plane has been chosen using ZEN 3.6. and is a compromise between the complete representation of the cell and the amount of work. The latter is characterized by the duration of the acquisition, which in turn depends on the spacing of the Z-planes.

On the one hand the light blue outline of the objects (microglia) allowed the signal strength to be assessed as well and the background error to be minimized. On the other hand, with the complete object and background mask, the ratio of the area occupied by the microglia was determined by observation.

In the CA3 region of the hippocampus at days 3, 7, and 14 after preparation, the mouse line had no significant effect on the percentage of area of microglia from the total image area (Table 3) despite morphological differences were noted by the observer. Thus, optical differences in microglial morphology have rather to be determined based on the shape of the microglia. Thus, no differences occurred regarding the total areas measured despite different morphological appearance (Figures 7E,F). However, the number of microglia of the TNF.MK.413 mice was lower with fewer projections than in the wild-type mice.

**TABLE 3:** Microglia: percentage area.

Mouse line	Time	CA3
Wild type	Day 3	21.32
	Day 7	29.62
	Day 14	9.17
TNF.MK.41.3	Day 3	14.45
	Day 7	26.81
	Day 14	15.28

### 3.4 Microglia: cell count

To assess microglia more precisely, their number per total image area was also determined (Table 4). Thus, at day 14 a significant effect on the number of microglia per maximum intensity projection with *p* < 0.0001 was notable. At this time point, there were significantly more microglia per total image in the wild-type compared to the TNF.MK.41.3 mice with *p* = 0.0450.

**TABLE 4:** Microglia: cell count.

Mouse line	Time	CA3
Wild type	Day 3	14.42
	Day 7	23.40
	Day 14	24.45
TNF.MK.41.3	Day 3	13.42
	Day 7	25.50
	Day 14	15.74

### 3.5 Microglia: number of projections

To describe the morphology more precisely, a parameter was developed to determine the activity state of the microglia. Here, the number of projections per microglia was calculated using the program “apeer.” In region CA3 at day 3 the count of process of the microglia of the wild-types was twice as high as the number of projections of the TNF-overexpressing mice. Till day 14 the count of process of these mouse lines were reduced compared to day 7 at both mice lines (Table 5).

**TABLE 5:** Microglia: number of projections.

Mouse line	Time	CA3
Wild type	Day 3	3.60
	Day 7	4.49
	Day 14	2.01
TNF.MK.41.3	Day 3	1.81
	Day 7	2.66
	Day 14	2.44

### 3.6 Microglia: percentage of nucleus area

**FIGURE 8:**
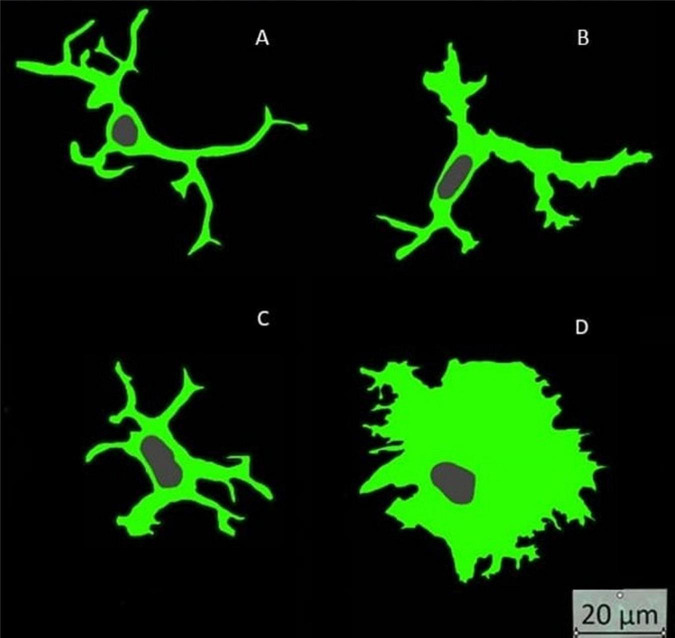
Microglia activity states: **(A)** ramified = spherical cell body at rest; **(B)** hyper ramified = active microglia with long processes; **(C)** bushy = active microglia with short processes and broad cytoplasm; **(D)** amoeboid = active microglia with broad and extensive cytoplasm.

Immunofluorescence images were randomly selected to morphologically subdivide Iba1-labeled microglia into their 4 activity states under qualitative visual inspection of the observer. The morphological differences were based on the work of Crews and Vetreno (2016), where microglia were classified into their 4 activity states based on their morphology. To further assess morphology and activity state, the area fraction of microglia nuclei from the total image area was determined (Table 6). This relationship between activity state and nuclear morphology was already postulated in a previous study (Crews and Vetreno, 2016). Likewise, rod cells can be identified additionally. In the present study, 197 microglia from randomly selected images were categorized into the four activity groups. Fourteen microglia could be assigned to the ramified group, 102 to the hyper-ramified group, 68 to the bushy group and 13 to the amoeboid group (Figure 8).

**TABLE 6:** Analysis of the relative values of the nucleus area of microglia.

Activity states	N	Mean value	Lower 95% CI	Upper 95% CI
Ramified	14	0.20	0.15	0.25
Hyper ramified	102	0.16	0.15	0.18
Bushy	68	0.41	0.39	0.44
Amoeboid	13	0.70	0.59	0.81

The aim of this dataset was to determine the morphology of microglia based on the area of the nucleus. A parametric analysis was performed with the data. The relative area of microglial nuclei was calculated for each of the four activity states. The associated 95% confidence intervals form the basis for subsequent upper and lower limits to classify microglia into their activity states based on the morphology of the nucleus. Table 6 shows the 95% confidence intervals of the four activity states:

This shows that microglia can be divided into 3 activity stages: ramified combined with hyper-ramified, bushy and amoeboid. The Kruskal-Whallis test with the subsequent pairwise comparisons of the four activity states with each other confirmed the result of the analysis of variance. All activity states were significantly different except for the microglia activity states ramified and hyper-ramified (*p* = 0.4581).

Applying this new algorithm, at days 3, 7, and 14 in region CA3, the mouse line had a significant effect on the area fraction of microglia nuclei (*p* = 0.0104, *p* = 0.0031, and *p* < 0.0001, respectively) (Table 7). This shows that the activity state of the microglia changes over time in the CA3 gene region in both mouse lines.

**TABLE 7:** Microglia: percentage of cell core area.

Mouse line	Time	CA3
Wild type	Day 3	0.21
	Day 7	0.22
	Day 14	0.21
TNF.MK.41.3	Day 3	0.17
	Day 7	0.18
	Day 14	0.16

## 4 Discussion

The aim of this study was to establish an unbiased AI-based scoring system for neuroglial cells that enables to assess changes in the morphology of neurons, astrocytes and microglia and to correlate this with their functionality. This was validated by analyzing areas of neuronal processes, astrocytes and microglia as well as the activity status of microglia in the hippocampus of TNF-overexpressing and wt mice as a model system. This scoring system was applied to TNFtg mice mimicking a proinflammatory status in the brain and can now be applied for a wide range of morphological changes, e.g. their spatial changes under epileptogenic influence (Cepeda et al., 2015; Sipe and Moore, 1976; Tani et al., 1973). The quality of an image depends on sample preparation, imaging and image processing. Sample preparation includes also preparation and immunofluorescence staining. By modifying the method for preparing organotypic hippocampal slice cultures (Galimberti et al., 2006; Stoppini et al., 1991), a shorter preparation time was during hippocampal isolation thereby achieving high quality slices. It is then possible to examine multiple Z subvolumes from one section (Kozlowski and Weimer, 2012). The smaller the spacing of the Z planes the more accurate the result values and the higher the time required, but this is negligible in case of an automated process (Plog et al., 2014). If the spacing of the Z-planes is chosen too large, minimal curvatures can be lost (Elias et al., 1971). In the present study, the choice of Z-plane spacing resulted in good distinction between object and background. By tiling the navigation image it was possible to get as many images as possible from one slice under visual control. This made it possible to reduce the number of mice while taking the 3-R principle into account. If one chooses a method that uses the intensity of the fluorescence for object recognition, a lack of structural information must be reduced by algorithms (Bjornsson et al., 2008). Thresholds can be used to reduce background error by assigning pixels that are in too small groups together (<150) to the background rather than counting them as objects (Kozlowski and Weimer, 2012; Plog et al., 2014). This was done in image processing considering pixel intensities, so that reliable IF measurements were guaranteed. One main advantage is the computerized procedure, equipped with artificial intelligence that reduced the subjective error by an observer and minimized the time factor. It should be noted that the evaluation was nevertheless carried out under personal control in order to train and assess the procedure. For this purpose, the mask of the evaluation program was used, which marked the object and background in its entirety (Figure 5) and was displayed for a few seconds during the measurement process.

When analyzing neuronal processes it must be taken into account that the highest density of neurofilament is found in long myelinated axons and long dendrites and can be most clearly visualized by immunofluorescence (Friede and Samorajski, 1970; Potter, 1971; Smith, 1973; Wuerker and Kirkpatrick, 1972; Wuerker and Palay, 1969). In contrast, signal strength is lower in short non-myelinated axons and perikarya because the density of neurofilament is lower (Peters, 1976; Wuerker and Kirkpatrick, 1972). Thus, the signal strength of immunofluorescence depends not only on the protocol and performance, but also on the nature of the tissue. Assuming that the hippocampi have the same origin, the tissue characteristics should be the same within the mouse lineage. The fact that the TNF-overexpressing mice had the largest area of the total image area should mean that the TNF levels must (at least indirectly) lead to an increase in the area of neuronal processes probably indicating higher excitability/functionality. However, this was reversible during the observed time course. Thus, it can be hypothesized that increased neuronal activity is related to changes in the neuronal network over the time. This was confirmed in functional studies of the hippocampus of the same mouse strain (Herkommer, 2023).

To examine astrocyte morphology, a system was developed to assess the morphological changes of the network of astrocytes based on the area fraction from the total image area. Nevertheless, it is important to note that although GFAP is cell specific for astrocytes compared to other neuroglial cells, its expression differs in different phenotypes (Cahoy et al., 2008). The protoplastic astrocyte for example is located in the gray matter and has finely branched projections in the neuropil. Another example, the fibrillar type, is found in the white matter and has many long filamentous projections. Thus, at all-time points and in both mouse lineages, a heterogeneous network was detected, which argues for different morphological subpopulations of astrocytes (Emsley and Macklis, 2006). The spatial organization of the projections remained the same, but an increase in the area of the astrocytes was measured at all-time points and in both mouse lineages. The increase in area has been observed previously in neurological diseases such as epilepsy, trauma and neurodegenerative diseases (Oberheim et al., 2008; Sofroniew, 2009; Wilhelmsson et al., 2006). Thus, on the one hand, this increase in area may be due to hypertrophy of the cell body and the projection thickness of astrocytes (Hirz, 2017); on the other hand, the number of astrocytes could have been increased or the ratios of the astrocyte subpopulation (Emsley and Macklis, 2006). However, based on the result without visual inspection, it was not possible to determine which point is true. When the area fraction of astrocytes is reduced cell death of astrocytes by apoptosis can be excluded because astrocytes appear to be rather resistant in this regard (Barkho et al., 2006; Hirz, 2017). Thus, a reduced area fraction must result from astrocyte projections becoming smaller or reproducing, or from a decrease in nucleus size. Since the area fraction of astrocytes of the TNF.MK.41.3-mice decreased again by day 14, it could be assumed that at least part of the changes in the astrocyte network were reversible (Wilhelmsson et al., 2006). Thus, the data indicate astrocyte activation, but which type of activation needs to assessed further. In the TNFtg mice it has already been shown that astrocyte activation appears as an unusual hypertrophy and hyperplasy of GFAP-positive astrocytes, especially after additional insults such as neurotropic virus infection (Hirz, 2017; Kramer et al., 2012).

For further analyses of the activation status, a system needs to be developed that separately record the individual characteristics of reactive astrocytes and those of the resting astrocytes and the possibility to merge the criteria. A system that could characterize the astrocytes more specifically by examining multiple cell parameters can complement the optical assessment. Thus, the cell parameters of microglia from this work could be applied to astrocytes in the future. However, due to their dense network, astrocytes could not yet be separated sufficiently with the available means to adopt the system of microglia. Reducing the layer thickness or the number of layers, might reduce the signal-positive pixels, but the 3D representation would also have been lost. Therefore, an algorithm needs to be developed that could distinguish between the process and the cell body, e.g., to detect the size of the cell bodies and then measure the processes individually and add the process width. In this way, the morphology of astrocytes could be exactly studied in the most accurate way and can be correlated to their activation status.

Since the area of microglia changes with their state of activity, it was hypothesized that the state of activity can be predicted based on the area fraction that microglia occupy in the maximum projection. Microglia are activated by many mediators (Kettenmann et al., 2011) and this was mimicked in this study by transgenic TNF overexpression (TNF.MK.41.3 mice). Accordingly, the area fraction of microglia was expected to be highest in TNF.MK.41.3 mice (Herkommer, 2023). In this study, there was no significant effect of the different mice strains on the area fraction of microglia. This means that the cell nucleus area and or the process number/size must have reached their maximum and indicate activation/hyperplasisa during culturing. Otherwise, one parameter could cancel out the other in their area. Thus, the area fraction as sole parameter of microglia cannot be enough to adequate characterize the morphology and activity state. Hovens et al. (2014) and further work based on Hovens uses a combination of microglia parameters like cell count, the size of process and the ratio of cell body to total cell size for assessment (Herkommer, 2023). It should also be noted that some of the microglia data published were gained from cell cultures and thus were not under the influence of the other neuroglial cells present in the brain/OHCs. Finally, regarding microglia area fraction, this parameter has the strongest significance when used in combination with the other parameters (see below).

For microglia cell count the new system by counting nuclei of microglia was proven useful. The nucleus detection step as the basis of nucleus counting was also applied in our group in a study on microglial cell cultures, but it based on more single steps (Hovens et al., 2014). Such additional steps were not necessary in this study due to the use of the artificial intelligence implemented in the measurement program. By this analysis, significant differences between the two mouse lines were observed which most likely indicate a different microglia cell number. If the number of microglia was combined with the number of microglial projections in the wild-type mice, on days 3 and 7 predominantly resting microglia were detected in the hippocampus. This is based on the fact that the microglia of the wild-types with an average number of 4.49 projections can be assigned to the resting microglia phenotype. The study by Onkels used the area of the projections in μm in combination with the ratio of the cell body to the cell size instead of the number of projections and reached the same conclusion. In the TNF.MK.41.3 mice, the activity state as determined by microglial process number was not significantly different, but the number of cells was significantly higher on day 7 than on day 3. Thus, the changes in microglia do account for their morphological changes and for their cell number. In conclusion, the parameter of microglial cell number is important to assess the microglia in their morphological changes, because the effect of the mouse line was highly significant at all three different fixation time points. The microglia cell count thus gives an indication of how many active or resting cells are present in an image.

It is well known that the resting “ramified” phenotype has many long, branched, and thin projections (Perry and Gordon, 1988; Rio-Hortega, 1932). The more active the microglia, the shorter and thicker the projections and the fewer the number of projections (Davis et al., 1994; Herkommer, 2023; Hovens et al., 2014; Ransohoff and Perry, 2009). Thus, the projections are a building block of the microglia phenotype that allows them to be classified in their activity state (Perry and Gordon, 1988; Rio-Hortega, 1932). However, in the previous study, the area of the projections was used instead of the number of projections, The area of projections did not show any significant differences between the TNF.MK.41.3- mouse line and the wild-type mice in the hippocampus. Relating these results to the associated activity state indicate that microglia with a low number of processes and thus high activity were obtained in mice that had higher TNF levels. The wild-type mice, on the other hand, with an average number of 4.49 projections could be assigned to the resting microglia phenotype (Rio-Hortega, 1932). In contrast to the microglia of wild-type mice, the microglia of TNF overexpressing mice possessed an overall lower number of projections. Here, it is also clear that TNF promotes the activity state of microglia. In this proinflammatory milieu, the likelihood of an epileptic seizure is significantly increased (Benson et al., 2015; De Simoni et al., 2000).

When evaluating the microglial processes located in the zone of influence around the nucleus not every process can be determined, because not every process was determined by the random Z-plane. The error is of course minimized by the addition of 30 levels. This might be reduced further by further reducing the Z-plane spacing, but this would increase the computation time of the algorithm. The problem of cell separation can be solved by defining the cell nuclei as black pixels with a green border as done in the present study. The AI-assisted evaluation system also allowed the detection of projections that had less intense staining. Especially, since the combination of the parameters of cell number, area fraction and area of nuclei with the number of projections can describe the microglia in their morphology. Furthermore, all the parameters can be determined in one calculation process, which reduces sources of error.

The morphological differences were based on the work of Crews and Vetreno (2016), where microglia were classified into their 4 activity states based on their morphology including also nuclear morphology (Crews and Vetreno, 2016). The aim was to create an algorithm, under optical control, which distinguishes as many activity states of the microglia as possible. The minimum would be the classification of the microglia into the two groups resting and activated. Each activity state could be assigned a mean value of the area of the cell nucleus in relation to the total area of the image. Because the groups ramified coupled with hyper-ramified, bushy and amoeboid differ significantly, it is possible to deduce 3 different activity states from the area of the microglial cell nuclei. The advantage of this classification is the possibility to develop a standardized procedure to determine the microglia activity states without visual inspection in a short time. However, when assessing the relative mean values of the nuclear area of each individual maximum projection, the values for both mouse lines were between 0.15 and 0.26% at each time point. However, this is not consistent with the observations and measurements of the previous microglial cell parameters. Thus, by calculating the mean value from the maximum projection, the activity state of the individual microglia may have been lost. Therefore, a system must be developed in the future that immediately assigns each individual microglia to one of the three groups without calculating an average value of a maximum projection per image. Thus, the percentage of microglia counted would have to be assigned to the activity state based on the nuclear area of each individual cell.

In summary, an AI-base scoring system for neuroglial cells was successfully established and applied in organotypic slice culture of TNF overexpressing and wild-type mice. Therefore, the reduction in the number of test animals and the short preparation time are resulting in good tissue quality. In addition, the basic tissue structure is preserved and a statement can be made about the 3-dimensional structural morphology of cells. Morphological differences were precisely analyzed but needs to be interpreted by the respective observer. The fact that the TNF-overexpressing mice had the largest area of total image area of neuronal process should mean that TNF levels (at least indirectly) indicate increased excitability/functionality. However, this was reversible during the observed time course. Therefore, it can be hypothesized that the changes in neuronal activity are associated with morphological changes of the neuronal process. Since the area of astrocytes in the total image area showed no clear changes over time and between mice, a system should be developed that uses several cell parameters to characterize the morphological changes of astrocytes. Thus, the cell parameters of microglia from this work could be applied to astrocytes in the future. In this way, the microglia parameters as a whole can provide information about the state of activity and how the cell count behaves. Regarding the evaluation, the ability to standardize the measurement by using the same algorithm is advantageous for large data sets. Furthermore, obtaining several measurement results of different cell parameters in one measurement process, additionally minimizes the time factor. Above all, by using this scoring system, the link between morphology and functionality of cells can be established.

## Data Availability

The raw data supporting the conclusions of this article will be made available by the authors, without undue reservation.

## Footnotes

1
https://imagej.net/Fiji/Downloads

## References

[B1] Amaral D. G. (1978). A Golgi study of cell types in the hilar region of the hippocampus in the rat. *J. Comp. Neurol.* 182 851–914. 10.1002/cne.901820508 730852

[B2] Barkho B. Song H. Aimone J. Smrt R. Kuwabara T. Nakashima K. (2006). Identification of astrocyte-expressed factors that modulate neural stem/progenitor cell differentiation. *Stem. Cells Dev.* 15 407–421. 10.1089/scd.2006.15.407 16846377 PMC2777811

[B3] Benson M. Manzanero S. Borges K. (2015). Complex alterations in microglial M1/M2 markers during the development of epilepsy in two mouse models. *Epilepsia* 56 895–905. 10.1111/epi.12960 25847097

[B4] Bjornsson C. Lin G. Al-Kofahi Y. Narayanaswamy A. Smith K. Shain W. (2008). Associative image analysis: A method for automated quantification of 3D multi-parameter images of brain tissue. *J. Neurosci. Methods* 170 165–178. 10.1016/j.jneumeth.2007.12.024 18294697 PMC2700351

[B5] Cahoy J. Emery B. Kaushal A. Foo L. Zamanian J. Christopherson K. (2008). A transcriptome database for astrocytes, neurons, and oligodendrocytes: A new resource for understanding brain development and function. *J. Neurosci.* 28 264–278. 10.1523/JNEUROSCI.4178-07.2008 18171944 PMC6671143

[B6] Caroni P. (1997). Overexpression of growth-associated proteins in the neurons of adult transgenic mice. *J. Neurosci. Methods* 71 3–9. 10.1016/s0165-0270(96)00121-5 9125370

[B7] Cepeda C. Chang J. Owens G. Huynh M. Chen J. Tran C. (2015). In Rasmussen encephalitis, hemichannels associated with microglial activation are linked to cortical pyramidal neuron coupling: A possible mechanism for cellular hyperexcitability. *CNS Neurosci. Ther.* 21 152–163. 10.1111/cns.12352 25438677 PMC4303544

[B8] Crews F. Vetreno R. (2016). Mechanisms of neuroimmune gene induction in alcoholism. *Psychopharmacology (Berl)* 233 1543–1557. 10.1007/s00213-015-3906-1 25787746 PMC4828484

[B9] Crews F. Nixon K. Wilkie M. (2004). Exercise reverses ethanol inhibition of neural stem cell proliferation. *Alcohol* 33 63–71. 10.1016/j.alcohol.2004.04.005 15353174

[B10] Davis E. Foster T. Thomas W. (1994). Cellular forms and functions of brain microglia. *Brain Res. Bull.* 34 73–78. 10.1016/0361-9230(94)90189-9 8193937

[B11] de Gracia P. Gallego B. Rojas B. Ramírez A. de Hoz R. Salazar J. (2015). Automatic counting of microglial cells in healthy and glaucomatous mouse retinas. *PLoS One* 10:e0143278. 10.1371/journal.pone.0143278 26580208 PMC4651327

[B12] De Paola V. Arber S. Caroni P. (2003). AMPA receptors regulate dynamic equilibrium of presynaptic terminals in mature hippocampal networks. *Nat. Neurosci.* 6 491–500. 10.1038/nn1046 12692557

[B13] De Simoni M. Perego C. Ravizza T. Moneta D. Conti M. Marchesi F. (2000). Inflammatory cytokines and related genes are induced in the rat hippocampus by limbic status epilepticus. *Eur. J. Neurosci.* 12 2623–2633. 10.1046/j.1460-9568.2000.00140.x 10947836

[B14] Droz B. Koenig H. Biamberardino L. Di Giamberardino L. (1973). Axonal migration of protein and glycoprotein to nerve endings. I. Radioautographic analysis of the renewal of protein in nerve endings of chicken ciliary ganglion after intracerebral injection of (3H)lysine. *Brain Res.* 60 93–127. 10.1016/0006-8993(73)90852-4 4126751

[B15] Elias H. Hennig A. Schwartz D. (1971). Stereology: Applications to biomedicalresearch. *Physiol. Rev.* 51 158–200. 10.1152/physrev.1971.51.1.158 4924033

[B16] Emsley J. Macklis J. (2006). Astroglial heterogeneity closely reflects the neuronal-defined anatomy of the adult murine CNS. *Neuron Glia Biol.* 2 175–186. 10.1017/S1740925X06000202 17356684 PMC1820889

[B17] Faustmann P. Haase C. Romberg S. Hinkerohe D. Szlachta D. Smikalla D. (2003). Microglia activation influences dye coupling and Cx43 expression of the astrocytic network. *Glia* 42 101–108. 10.1002/glia.10141 12655594

[B18] Feng G. Mellor R. Bernstein M. Keller-Peck C. Nguyen Q. Wallace M. (2000). Imaging neuronal subsets in transgenic mice expressing multiple spectral variants of GFP. *Neuron* 28 41–51. 10.1016/s0896-6273(00)00084-2 11086982

[B19] Franke W. (1987). Nuclear lamins and cytoplasmic intermediate filament proteins: A growing multigene family. *Cell* 48 3–4. 10.1016/0092-8674(87)90345-x 3791413

[B20] Friede R. Samorajski T. (1970). Axon caliber related to neurofilaments and microtubules in sciatic nerve fibers of rats and mice. *Anat. Rec.* 167 379–387. 10.1002/ar.1091670402 5454590

[B21] Gähwiler B. (1981). Organotypic monolayer cultures of nervous tissue. *J. Neurosci. Methods* 4 329–342. 10.1016/0165-0270(81)90003-0 7033675

[B22] Gähwiler B. Capogna M. Debanne D. McKinney R. Thompson S. (1997). Organotypic slice cultures: A technique has come of age. *Trends Neurosci.* 20 471–477. 10.1016/s0166-2236(97)01122-3 9347615

[B23] Galimberti I. Gogolla N. Alberi S. Santos A. Muller D. Caroni P. (2006). Long-term rearrangements of hippocampal mossy fiber terminal connectivity in the adult regulated by experience. *Neuron* 50 749–763. 10.1016/j.neuron.2006.04.026 16731513

[B24] Hailer N. Grampp A. Nitsch R. (1999). Proliferation of microglia and astrocytes in the dentate gyrus following entorhinal cortex lesion: A quantitative bromodeoxyuridine-labelling study. *Eur. J. Neurosci.* 11 3359–3364. 10.1046/j.1460-9568.1999.00808.x 10510203

[B25] Herkommer L. (2023). *Influence of tumor necrosis factor (TNF) and its signalling pathways on neuronal functions*. From the Institute of Veterinary Pathology and the Institute of Veterinary Physiology and Biochemistry at Justus Liebig University, Gießen.

[B26] Hinkerohe D. Smikalla D. Haghikia A. Heupel K. Haase C. Dermietzel R. (2005). Effects of cytokines on microglial phenotypes and astroglial coupling in an inflammatory coculture model. *Glia* 52 85–97. 10.1002/glia.20223 15920725

[B27] Hirz M. (2017). *Pathogenese Epileptiformer Krämpfe bei TNF-Transgenen Mäusen Nach Borna Disease Virus-Infektion.* Dissertation. Gießen: Justus-Liebig- Universität.

[B28] Hoffman P. Lasek R. (1975). The slow component of axonal transport. Identification of major structural polypeptides of the axon and their generality among mammalian neurons. *J. Cell. Biol.* 66 351–366. 10.1083/jcb.66.2.351 49355 PMC2109569

[B29] Hovens I. Nyakas C. Schoemaker R. G. (2014). A novel method for evaluating microglial activation using ionized calcium-binding adaptor protein-1 staining: Cell body to cell size ratio. *Neuroimmunol Neuroinflammation* 1:82. 10.4103/2347-8659.139719

[B30] Kettenmann H. Hanisch U. Noda M. Verkhratsky A. (2011). Physiology of microglia. *Physiol. Rev.* 91 461–553. 10.1152/physrev.00011.2010 21527731

[B31] Kozlowski C. Weimer R. (2012). An automated method to quantify microglia morphology and application to monitor activation state longitudinally in vivo. *PLoS One* 7:e31814. 10.1371/journal.pone.0031814 22457705 PMC3294422

[B32] Kramer K. Schaudien D. Eisel U. Herzog S. Richt J. Baumgärtner W. (2012). TNF-overexpression in Borna disease virus-infected mouse brains triggers inflammatory reaction and epileptic seizures. *PLoS One* 7:e41476. 10.1371/journal.pone.0041476 22848506 PMC3405098

[B33] Lovelace M. Cahill D. M. A. (2007). rapid cell counting method utilising acridine orange as a novel discriminating marker for both cultured astrocytes and microglia. *J. Neurosci. Methods* 165 223–229. 10.1016/j.jneumeth.2007.06.009 17662460

[B34] Merbl Y. Sommer A. Chai O. Aroch I. Zimmerman G. Friedman A. (2014). Tumor necrosis factor-α and interleukin-6 concentrations in cerebrospinal fluid of dogs after seizures. *J. Vet. Intern. Med.* 28 1775–1781. 10.1111/jvim.12462 25308784 PMC4895630

[B35] Nixon K. Kim D. Potts E. He J. Crews F. (2008). Distinct cell proliferation events during abstinence after alcohol dependence: Microglia proliferation precedes neurogenesis. *Neurobiol. Dis.* 31 218–229. 10.1016/j.nbd.2008.04.009 18585922 PMC2680247

[B36] Oberheim N. Tian G. Han X. Peng W. Takano T. Ransom B. (2008). Loss of astrocytic domain organization in the epileptic brain. *J. Neurosci.* 28 3264–3276. 10.1523/JNEUROSCI.4980-07.2008 18367594 PMC6670598

[B37] Osei D. Baumgart-Vogt E. Ahlemeyer B. Herden C. (2024). Tumor necrosis factor-α receptor 1 mediates borna disease virus 1-induced changes in peroxisomal and mitochondrial dynamics in neurons. *Int. J. Mol. Sci.* 25:1849. 10.3390/ijms25031849 38339126 PMC10855776

[B38] Perry V. Gordon S. (1988). Macrophages and microglia in the nervous system. *Trends Neurosci.* 11 273–277. 10.1016/0166-2236(88)90110-5 2465626

[B39] Peters A. (1976). “The cellular sheaths of neurons,” in *The Fine Structure of the Nervous System: The Neurons and Supporting Cells.* Available online at: https://ci.nii.ac.jp/naid/10024505610/ (accessed November 10, 2022).

[B40] Plog B. Moll K. Kang H. Iliff J. Dashnaw M. Nedergaard M. (2014). A novel technique for morphometric quantification of subarachnoid hemorrhage-induced microglia activation. *J. Neurosci. Methods* 229 44–52. 10.1016/j.jneumeth.2014.04.001 24735531 PMC4077584

[B41] Potter H. (1971). The distribution of neurofibrils coextensive with microtubules and neurofilaments in dendrites and axons of the tectum, cerebellum and pallium of the frog. *J. Comp. Neurol.* 143 385–409. 10.1002/cne.901430402 4110014

[B42] Ransohoff R. Perry V. (2009). Microglial physiology: Unique stimuli, specialized responses. *Annu. Rev. Immunol.* 27 119–145. 10.1146/annurev.immunol.021908.132528 19302036

[B43] Rio-Hortega (1932). “Mikroglia,” in *Zytologie und Zellpathologie des Nervensystems, Bd. 2*, ed. Herausgeber P. W. (New York: PB. Hoeber), 483–534.

[B44] Schlaepfer W. (1971). Vincristine-induced axonal alterations in rat peripheral nerve. *J. Neuropathol. Exp. Neurol.* 30 488–505. 10.1097/00005072-197107000-00012 4327885

[B45] Sipe J. Moore R. (1976). Astrocytic gap junctions in the rat lateral hypothalamic area. *Anat. Rec.* 185 247–251. 10.1002/ar.1091850211 1275310

[B46] Smeal R. Stewart K. Iacob E. Fujinami R. White H. Wilcox K. (2012). The activity within the CA3 excitatory network during Theiler’s virus encephalitis is distinct from that observed during chronic epilepsy. *J. Neurovirol.* 18 30–44. 10.1007/s13365-012-0082-5 22328242 PMC4397904

[B47] Smith R. (1973). Microtubule and neurofilament densities in amphibian spinal root nerve fibers: Relationship to axoplasmic transport. *Can. J. Physiol. Pharmacol.* 51 798–806. 10.1139/y73-123 4128420

[B48] Sofroniew M. (2009). Molecular dissection of reactive astrogliosis and glial scar formation. *Trends Neurosci.* 32 638–647. 10.1016/j.tins.2009.08.002 19782411 PMC2787735

[B49] Steinert P. Steven A. Roop D. (1985). The molecular biology of intermediate filaments. *Cell* 42 411–420. 10.1016/0092-8674(85)90098-4 2411418

[B50] Stoppini L. Buchs P. Muller D. (1991). A simple method for organotypic cultures of nervous tissue. *J. Neurosci. Methods* 37 173–182. 10.1016/0165-0270(91)90128-m 1715499

[B51] Takeuchi H. Jin S. Wang J. Zhang G. Kawanokuchi J. Kuno R. (2006). Tumor necrosis factor-alpha induces neurotoxicity via glutamate release from hemichannels of activated microglia in an autocrine manner. *J. Biol. Chem.* 281 21362–21368. 10.1074/jbc.M600504200 16720574

[B52] Tani E. Nishiura M. Higashi N. (1973). Freeze-fracture studies of gap junctions of normal and neoplastic astrocytes. *Acta Neuropathol.* 26 127–138. 10.1007/BF00697748 4357785

[B53] Verkhratsky A. Kirchhoff F. (2007). Glutamate-mediated neuronal-glial transmission. *J. Anat.* 210 651–660. 10.1111/j.1469-7580.2007.00734.x 17504269 PMC2375757

[B54] Vienenkoetter J. Eisel U. L. M. Culmsee C. Hirz M. Herden C. (2015). “Role of Lipocalin 2 in Virally Induced Neuroinflammatory Processes by the Use of BDV Infected TNF-Transgenic and TNF Receptor k.o.-Mice. Deutsche Gesellschaft für Neuropathologie und Neuroanatomie. Unter Mitarbeit von Deutsche Gesellschaft Für Neuropathologie Und Neuroanatomie,” *60th Annual Meeting of the German Society for Neuropathology and Neuroanatomy (DGNN), Berlin, 26.-28.08.2015*. Düsseldorf: German Medical Science GMS Publishing House

[B55] Wilhelmsson U. Bushong E. Price D. Smarr B. Phung V. Terada M. (2006). Redefining the concept of reactive astrocytes as cells that remain within their unique domains upon reaction to injury. *Proc. Natl. Acad. Sci. U S A.* 103 17513–17518. 10.1073/pnas.0602841103 17090684 PMC1859960

[B56] Wuerker R. Kirkpatrick J. (1972). Neuronal microtubules, neurofilaments, and microfilaments. *Int. Rev. Cytol.* 33 45–75. 10.1016/s0074-7696(08)61448-5 4562606

[B57] Wuerker R. Palay S. (1969). Neurofilaments and microtubules in anterior horn cells of the rat. *Tissue Cell.* 1 387–402. 10.1016/s0040-8166(69)80012-1 18631475

[B58] Yokose J. Ishizuka T. Yoshida T. Aoki J. Koyanagi Y. Yawo H. (2011). Lineage analysis of newly generated neurons in organotypic culture of rat hippocampus. *Neurosci. Res.* 69 223–233. 10.1016/j.neures.2010.11.010 21145363

[B59] Zhao X. Ahram A. Berman R. Muizelaar J. Lyeth B. (2003). Early loss of astrocytes after experimental traumatic brain injury. *Glia* 44 140–152. 10.1002/glia.10283 14515330

